# News and Miscellany

**Published:** 1900-07

**Authors:** 


### News and Miscellany.

**Dr. R. A. Goethe,** of Boerne, Texas, is attending the New York Polyclinic.

---

**Change of Address:** Dr. E. L. Wedemeyer, from Buckhorn, Texas, to Gay Hill, Texas.

---

**The American Medical Association Medal,** a gold disc about the size of a twenty-dollar gold piece and double the thickness, handsomely engraved, was by unanimous vote of the committee awarded to Dr. A. L. Benedict, of Buffalo, N. Y., the accomplished editor of *The American Therapist.* Dr. Benedict's paper was entitled "Quantitative Tests for Proteolysis." The Senn Medal was awarded to Dr. F. Gregory Connell, of Chicago. His essay was entitled "Exstrophy of the Bladder."

---

**Fraud Run to Earth.**—Chicago, Ill.—Government officials today invaded the Metropolitan Medical College, an alleged "diploma mill," at 866 West Van Buren street, and arrested the officers, President James Armstrong, Vice-President J. H. Randell, Secretary Thomas Armstrong and former Secretary Charles M. Hovey. The men are charged with having used the mails to defraud, and it is said their receipts amount to many thousands of dollars. Failing to give suitable bonds, all four went to jail. The institution with which the defendants are connected is also known as the Independent Medical College and the National Law School, and the evidence shows that its faculty has been selling degrees for the practice of medicine and law at prices ranging from $3 to $200, the compensation being determined by the amount the would-be professional men were willing to advance.

Postoffice Inspector Gould, who made the arrest after accumulating a lot of documentary evidence, says the fraud is one of the largest the postal authorities have ever had to deal with, and that the graduates of the institution are practicing in every State and even abroad. He says frequent complaints of malpractice have been made against the holders of the diplomas of the institution. Inspector Gould has a bunch of unsigned certificates of practice for the State of Texas, and he says the name of the District Clerk is forged to these for an extra compensation. Texas is said to have been the most fertile field for the "graduates" of the institution. The British Consul has made repeated efforts to run the Metropolitan faculty out of business. Great Britain is said to have been flooded with these diplomas. It is said that dozens of "graduates" of Armstrong's institutions are practicing in Indiana and in all parts of the world.—*Dallas News.*

---

**Medical Department,** University of Texas, Ninth Annual Commencement, Galveston. Graduates in School of Medicine: G. W. Allen, Jr., H. F. Blailock, B. S. Brown, H. B. Decherd, John H. Foster, Frank C. Gregg, J. E. Griffin, R. S. Jackscon, J. P. Lokey, O. H. Radkey, Charlotte M. Schaefer, T. E. Spaulding, Herbert Sterzing, Z. N. Thornton, T. F. Bryan, Ella Develin. School of Pharmacy: M. S. Ball, Miss Emma Domingo, D. P. English, H. J. Flavin, G. T. French, B. E. Gatewood, T. R. James, Frank McCullough, J. H. Perkins, C. L. Reynolds and W. L. Smith. Training School for Nurses: Miss Carolyn Bryan, Mrs. Winnie Champion, Miss Emilie Dirksen, Mrs. Marion Dunklin, Miss Minnie Ferguson, Miss Annie Lee Fitch, Miss Ada Horton, Miss Ella Rhodes, Miss Sallie Will Smith. Hospital Internes distinctions were won this year by Dr. John H. Foster of Georgetown, Dr. Frank C. Gregg of Manor, Dr. Charlotte M. Schaefer of San Antonio, Dr. B. S. Brown of Corsicana, Dr. H. B. Decherd of Austin and Dr. O. H. Radkey of Austin. Drs. Foster and Gregg "attained the distinction of reaching an average grade of 90 in the examinations."

At a subsequent meeting of the faculty the following changes were made in the teaching corps: Dr. W. L. Rogers, of Galveston, was appointed professor of ophthalmology, otology, etc.; Prof. Edward Randall (of the faculty), lecturer on physical diagnosis; Prof. Allen J. Smith, dean, professor of medicine and lecturer on nervous diseases; Dr. I. M. Cline, lecturer on climatology; Professor R. R. D. Cline, lecturer on pharmacy; Dr. Wm. Gammon, lecturer on skin and venereal diseases; Dr. Thomas Flavin, demonstrator of anatomy; Dr. Wm. Gammon, demonstrator of pathology; Dr. L. E. Magnenat, demonstrator of biology: Mr. Conn L. Milburn, demonstrator of chemistry; Dr. T. L. Kenedy, demonstrator of gynecology; Dr. Julius Ruhl, demonstrator of obstetrics; Dr. L. R. Dudgeon, demonstrator of surgery.

Page(s) missing

